# Defining the Potential Targets for Biological Activity of Isoegomaketone Based on Network Pharmacology and Molecular Docking Methods

**DOI:** 10.3390/life12122115

**Published:** 2022-12-15

**Authors:** Juzhao Zhang, Ruo Wang, Yuxuan Qin, Chengling Feng

**Affiliations:** Shanghai Jiao Tong University School of Medicine, Shanghai 200025, China

**Keywords:** isoegomaketone, network pharmacology, molecular docking, biological activity, active molecule

## Abstract

Isoegomaketone is a water-soluble natural ketone compound that is commonly present in *Rabdosia angustifolia* and *Perilla frutescens*. At present, it is known that isoegomaketone has a wide range of pharmacological activity, but there has been no thorough investigation of its potential targets. As a result, we examined the potential targets of isoegomaketone using the network pharmacology approach. In our study, the TCM Database@Taiwan was utilized to search for the chemical formula. The pharmacological characteristics of isoegomaketone were then evaluated in silico using the Swiss Absorption, Distribution, Metabolism, and Excretion (Swiss ADME) and Deep Learning–Acute Oral Toxicity (DL-AOT) methods, and the potential isoegomaketone target genes were identified using a literature study. Additionally, using the clusterProfiler R package 3.8.1, the Gene Ontology (GO) enrichment analysis and the Kyoto Encyclopedia of Genes and Genomes (KEGG) pathway enrichment analysis of target genes were performed. In order to obtain the protein interaction network, we simultaneously submitted the targets to the STRING database. After this, we performed molecular docking with respect to targets and isoegomaketone. Finally, we created visual networks of protein–protein interactions (PPI) and examined these networks. Our results showed that isoegomaketone had good drug-likeness, bioavailability, medicinal chemistry friendliness, and acceptable toxicity. Subsequently, through the literature analysis, 48 target genes were selected. The bioinformatics analysis and network analysis found that these target genes were closely related to the biological processes of isoegomaketone, such as atherosclerotic formation, inflammation, tumor formation, cytotoxicity, bacterial infection, virus infection, and parasite infection. These findings show that isoegomaketone may interact with a wide range of proteins and biochemical processes to form a systematic pharmacological network, which has good value for the creation and use of drugs.

## 1. Introduction

Traditional Chinese medicine contains a large number of active drug molecules [1]. Through the mining of traditional Chinese medicine, the development of drugs can be greatly enriched [2]. Isoegomaketone is a water-soluble natural ketone compound [3]. It is commonly present in traditional Chinese medicines *Rabdosia angustifolia* and *Perilla frutescens* [4]. Recent studies have shown that isoegomaketone has various types of biological activity, such as anti-inflammatory, anti-tumor, anti-rheumatoid arthritis, etc. [5,6,7,8,9]. Thus, isoegomaketone is considered to be an important active component of *Perilla frutescens* and has great drug development potential. However, the potential targets of isoegomaketone and its potential for further development are not yet fully understood. At present, the extraction of potential targets from existing research and the use of computer methods to predict the potential development directions has become the main method [10,11,12]. Compared with traditional methods, this method provides more convenient technology and a clearer direction for drug design and development.

As a result, we applied the network pharmacology approach to comprehensively examine the pharmacological effects of isoegomaketone. Firstly, the Swiss Absorption, Distribution, Metabolism, and Excretion (Swiss ADME) and Deep Learning–Acute Oral Toxicity (DL-AOT) methods were used to evaluate in silico the drug properties of isoegomaketone. Furthermore, we provided candidate target genes through literature analysis. Additionally, Gene Ontology (GO) enrichment analysis, Kyoto Encyclopedia of Genes and Genomes (KEGG) enrichment analysis, and molecular docking were performed on these potential target genes. Finally, we thoroughly demonstrated the probable targets of the compound by creating the isoegomaketone pharmacological interaction network.

## 2. Method

### 2.1. Molecular Formula and In Silico Drug Properties of Isoegomaketone

An important database resource that is free to use and readily available is the TCM Database@Taiwan (http://tcm.cmu.edu.tw/ (accessed on 25 March 2022)), which houses sources of chemical and pharmacological information on isoegomaketone [13]. The Swiss ADME (http://www.swissadme.ch/ (accessed on 25 March 2022)), which supports drug development, enables researchers to calculate the physicochemical descriptors and predict the ADME parameters, pharmacokinetic features, drug-like nature, and medicinal chemistry friendliness of one or more small compounds [14]. Thus, in our study, the in silico drug properties of isoegomaketone were analyzed using the Swiss ADME.

The DL-AOT prediction server (http://www.pkumdl.cn:8080/DLAOT/DLAOThome.php (accessed on 25 March 2022)) is a tool used to evaluate the acute oral toxicity (AOT) of small molecules [15]. Thus, in our study, the in silico toxicity (LD50) of isoegomaketone was analyzed using the DL-AOT prediction server.

### 2.2. Target Gene Screening for Isoegomaketone

All candidate potential targets are derived from literature analysis. Literature analysis was carried out through various academic databases, with the keyword “isoegomaketone” [5,6,16,17,18,19,20,21,22,23,24]. Finally, eleven published, peer-reviewed pharmacological studies on isoegomaketone were selected. All eleven selected pharmacological studies on isoegomaketone demonstrated high research quality and were all carried out via in vivo or in vitro methods.

### 2.3. Analysis of PPI Network

One of the online databases that compiles all publicly available sources of knowledge of PPI is STRING 11.0 (https://string-db.org/ (accessed on 29 March 2022)), which enables users to supplement the knowledge already known about PPI with computational predictions [25]. We uploaded 48 putative targets of isoegomaketone to the STRING database in order to build a PPI network, with the species defined as “Homo sapiens” and the minimum interaction score set to 0.4. After this, the outcomes were imported into Cytoscape 3.7.2 for visual evaluation. Cytoscape, an open-source software platform for visualizing complex networks, can calculate the parameters of each node in the network diagram, such as the degree, betweenness centrality (BC), and closeness centrality (CC) [26]. We utilized the cytoHubba plug-in topological method to identify the significant protein nodes and subnetworks in the network, after filtering the target nodes by the matching median values of degree values, BC and CC in the PPI network [27].

### 2.4. Gene Function and Pathway Enrichment Analysis

GO functional annotation analysis is a standard method used to carry out large-scale functional enrichment analyses of genes, and it comprises biological processes (BP), molecular functions (MF), and cellular components (CC) [28]. It is possible to assign functional meanings to genes and genomes at the molecular and higher levels by adopting the widely used Kyoto Encyclopedia of Genes and Genomes (KEGG) pathway [29]. Using the clusterProfiler R package 3.8.1 with FDR < 0.2 (FDR, false discovery rate) and *p* value < 0.05, KEGG pathway analysis and GO analysis were carried out, and the most important targets engaged in the pertinent biological processes were examined.

### 2.5. Compound–Target Molecular Docking

The 2D structure of isoegomaketone, downloaded from PubChem (https://pubchem.ncbi.nlm.nih.gov/ (accessed on 26 March 2022)) in mol2 format, was imported into the AutoDockTools 1.5.6 software. After checking its spatial structure, adding atomic charges, assigning atomic types, and changing all flexible bonds to rotatable by default, it was saved in pdbqt format as a docking ligand. The 3D structures of target-gene-associated proteins were downloaded from the Uniprot database (https://www.uniprot.org/ (accessed on 26 March 2022)) and the pdb database (https://www.rcsb.org/ (accessed on 26 March 2022)), and they were then saved in pdb format. AutoDockTools 1.5.6 software was used to carry out the removal of origin ligands, removal of water molecules, hydrogenation, charge addition, and various optimizations. The grid box was set as the default value and the final optimized proteins were saved in pdbqt format as docking acceptors. Molecular docking was performed using AutoDock Vina 1.1.2. The protein structure was set during molecular docking to be a rigid macromolecule, and the Genetic Algorithm Parameters algorithm was used. PyMOL was used to illustrate the outcomes for the group in which each protein had the lowest binding energy.

## 3. Result

### 3.1. Molecular Formula and In Silico Drug Properties of Isoegomaketone

**Figure 1:**
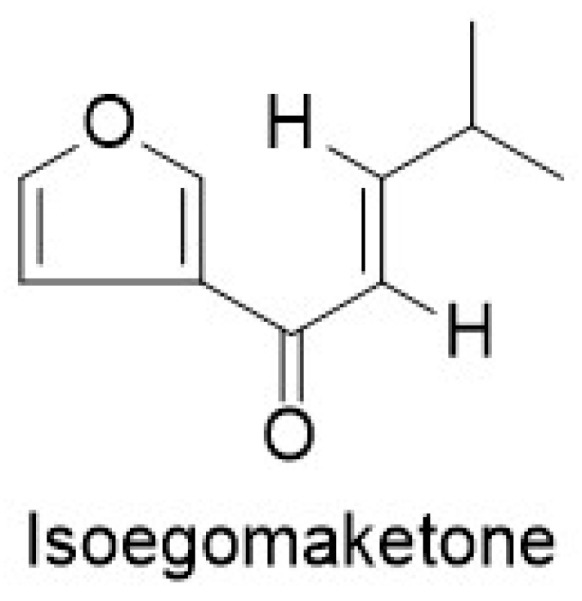
Molecular formula of isoegomaketone.

We obtained the chemical formula for isoegomaketone using the TCM Database@Taiwan database (Figure 1). Through Swiss ADME, we obtained essential ADME-related data on isoegomaketone, and the drug-likeness as well as the medicinal chemistry of isoegomaketone were subsequently evaluated via the classic formula. The results are provided in Table 1. Regarding the drug-likeness, lead-drug likeness, and medicinal chemistry friendliness, the pharmacokinetic characteristics of isoegomaketone were consistent with Lipinski’s rule of five, the Ghose filter, Veber’s Rule, and the RO (3) rule, and the synthetic accessibility was 2.9. Definitions of these rules and parameters can be found in References [30,31,32,33,34]. 

The LD50 value of isoegomaketone was predicted by DL-AOT, as shown in Table 2. The result showed that the LD50 value of isoegomaketone was 31.2 mg/kg, while the evaluation indicated “caution”.

### 3.2. Candidate Target Genes from Literature Analysis

Through literature analysis, eleven papers were selected. Finally, 48 candidate targets of isoegomaketone were selected for study, as shown in Table 3.

### 3.3. Construction of the PPI Network and Screening of Key Targets

**Figure 2:**
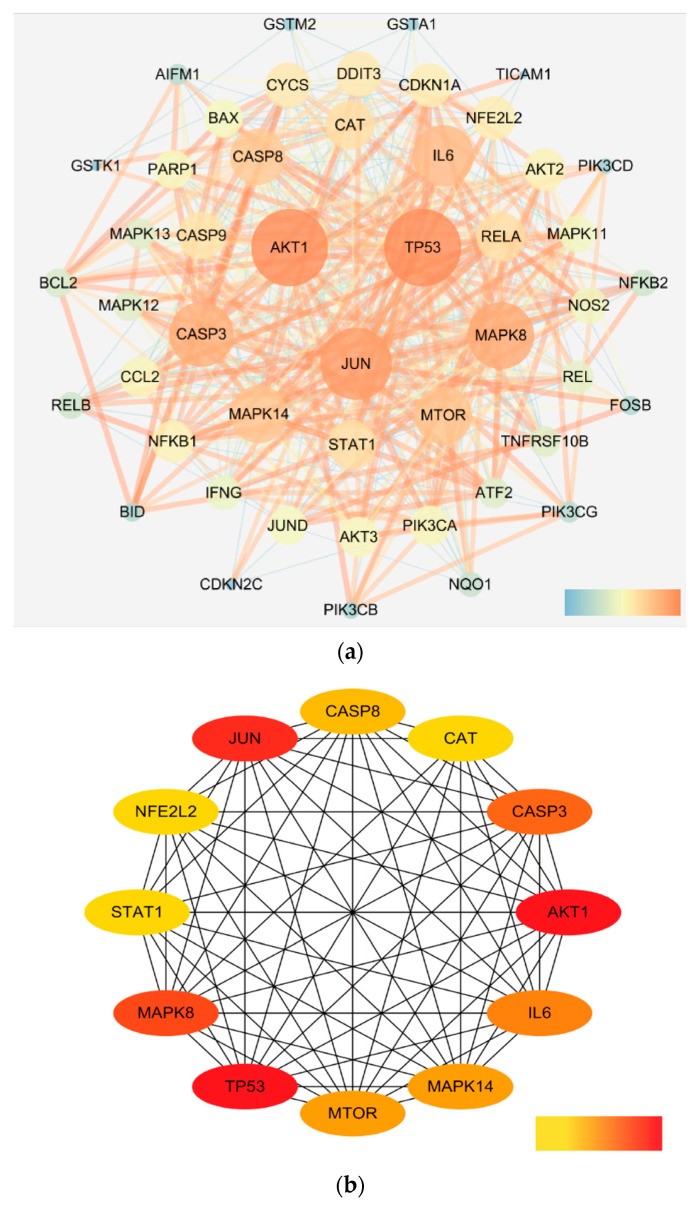
(**a**). Protein interaction network of 48 candidate targets. (**b**). Protein interaction network diagram of 12 key targets.

In order to obtain the PPI network association, we imported the 48 target genes into the STRING database and chose Homo sapiens as the organism. The free nodes were then eliminated, and the data were loaded into Cytoscape 3.7.2 for visualization (Figure 2a). The circle’s size and color varied with the degree value, while the width and color of the edges varied with the total score. The image shows 48 targets and 485 interactions, indicating that the more projected disease-related targets there are, the more likely it is that there are successful interactions between those targets. Calculated by the Network Analyzer plug-in in Cytoscape, the average closeness centrality (CC) was 0.63, the average betweenness centrality (BC) was 0.01, and the average degree value (DV) of the node was 19.1. Twelve of them had DV, BC, and CC values that were higher than the norm, while AKT1, TP53, JUN, MAPK8, CASP3, IL6, MTOR, and MAPK14 all displayed high connection degrees that were greater than 30. These could be the key target proteins that are the focus of isoegomaketone. The subnetwork of these key targets is shown in Figure 2b. Table S1 displays the specific node properties in detail.

### 3.4. GO Enrichment Analysis

**Figure 3:**
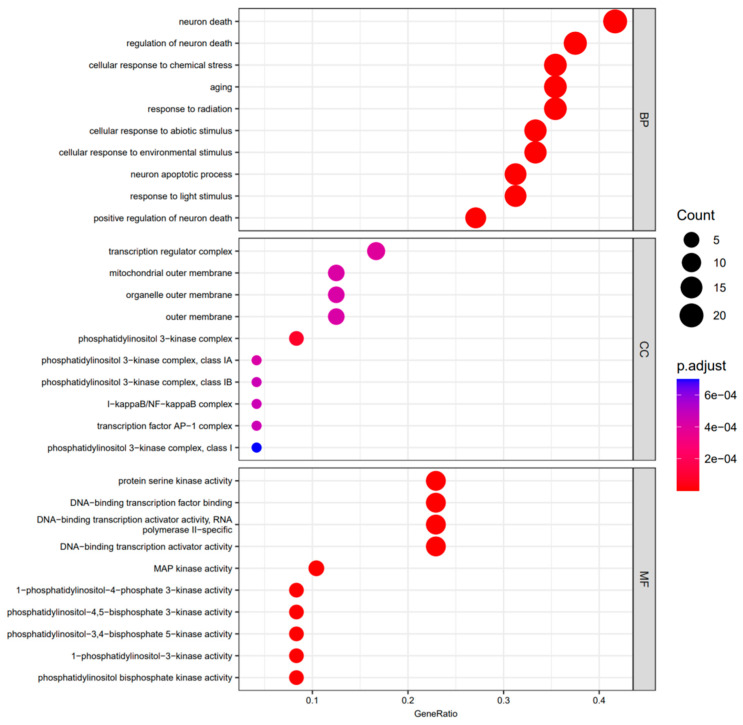
GO enrichment summary of the target genes. The results of biological process (BP), cellular component (CC), and molecular function (MF) are separately shown. The ratio of target genes is represented by the height of the bar.

We performed GO enrichment analysis on the 48 chosen target genes in order to further examine them. The majority of the target genes were identified as being engaged in biological processes (BP), cellular components (CC), and molecular functions (MF) according to GO enrichment analysis. BP enrichment mainly involved the following target genes: neuron death (20/48), regulation of neuron death (18/48), cellular response to chemical stress (17/48), aging (17/48), response to radiation (17/48), cellular response to abiotic stimulus (16/48), cellular response to environmental stimulus (16/48), neuron apoptotic process (15/48), response to light stimulus (15/48), and positive regulation of neuron death (13/48). The following target genes were mostly associated with CC enrichment: transcription regulator complex (8/48), mitochondrial outer membrane (6/48), organelle outer membrane (6/48), outer membrane (6/48), phosphatidylinositol 3-kinase complex (4/48) and protein–DNA complex (4/48). Target genes for MF enrichment included the following in particular: protein serine kinase activity (11/48), DNA-binding transcription factor binding (11/48), DNA-binding transcription activator activity (11/48), and MAP kinase activity (5/48). All the results are shown in Figure 3.

### 3.5. KEGG Enrichment Analysis

**Figure 4:**
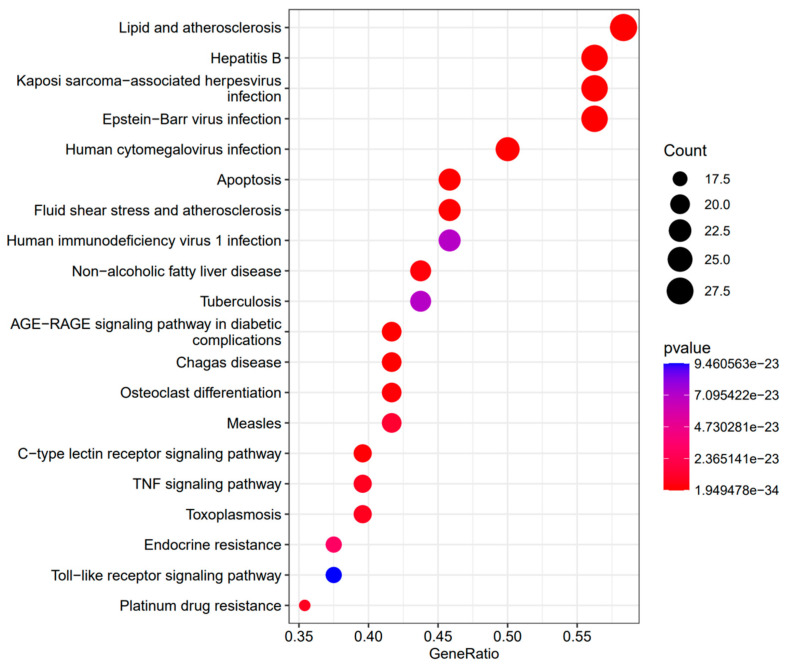
KEGG enrichment analysis of target genes.

We performed KEGG enrichment analysis of these target genes concurrently. KEGG pathway enrichment analysis revealed that 48 possible target genes were enriched and that there were significant relationships between the target genes and 194 signal pathways (FDR ≤ 0.05). The top 20 pathways with the highest enrichment ratios are demonstrated in Figure 4. The pathway with the largest count and lowest *p* value was the lipid and atherosclerosis pathway.

### 3.6. Results of Molecular Docking

**Figure 5:**
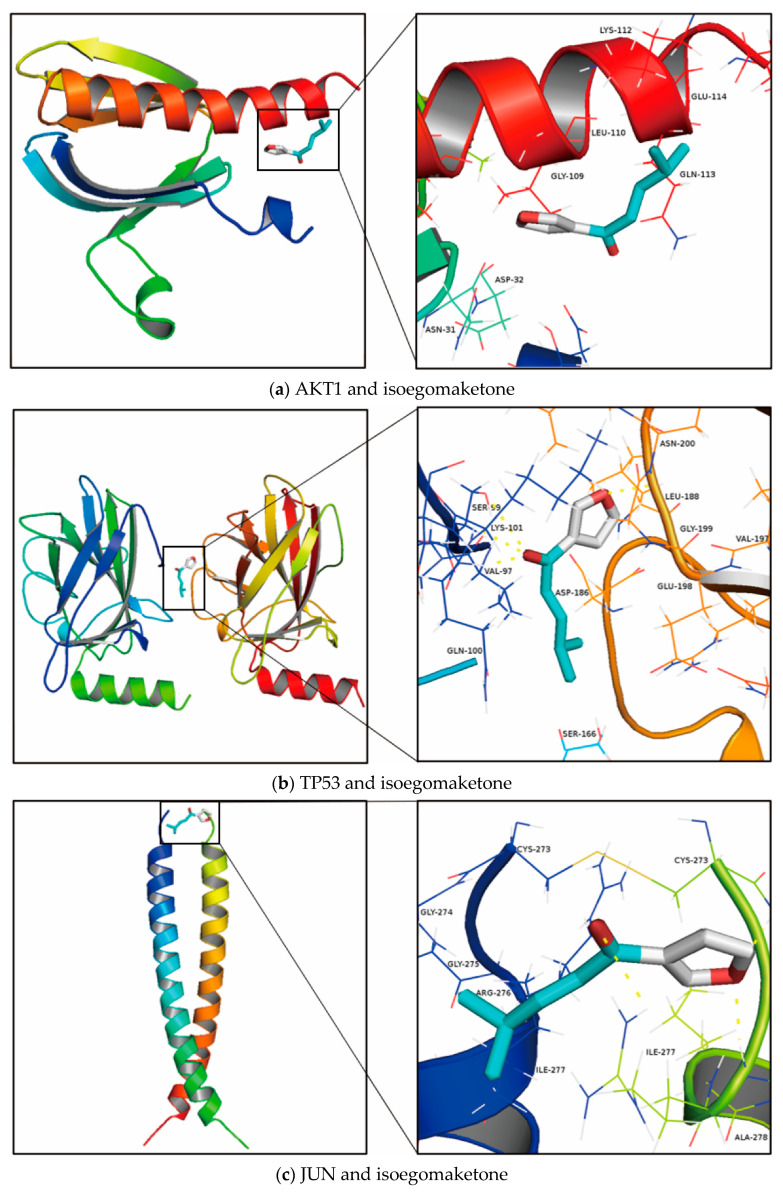

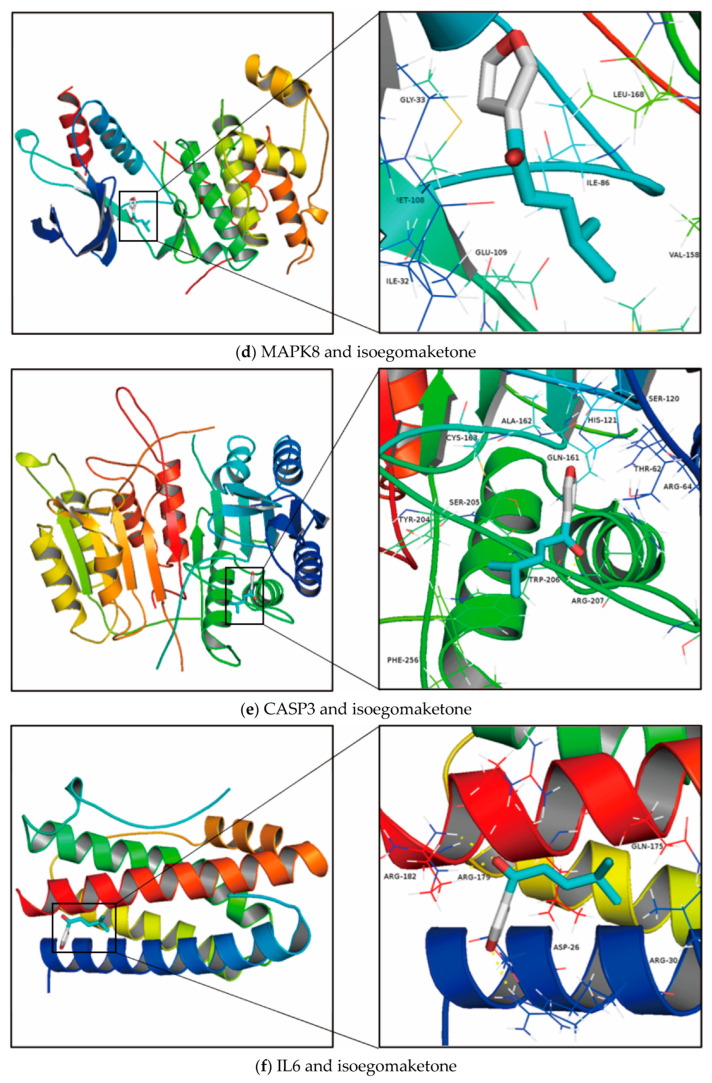

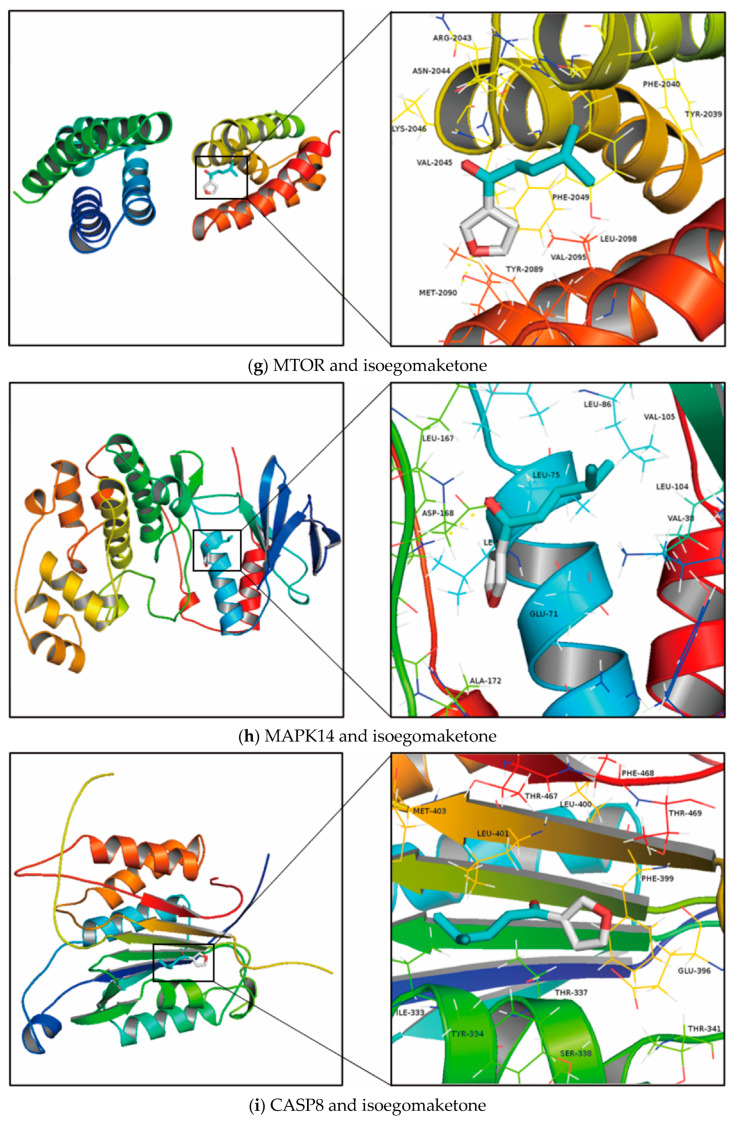

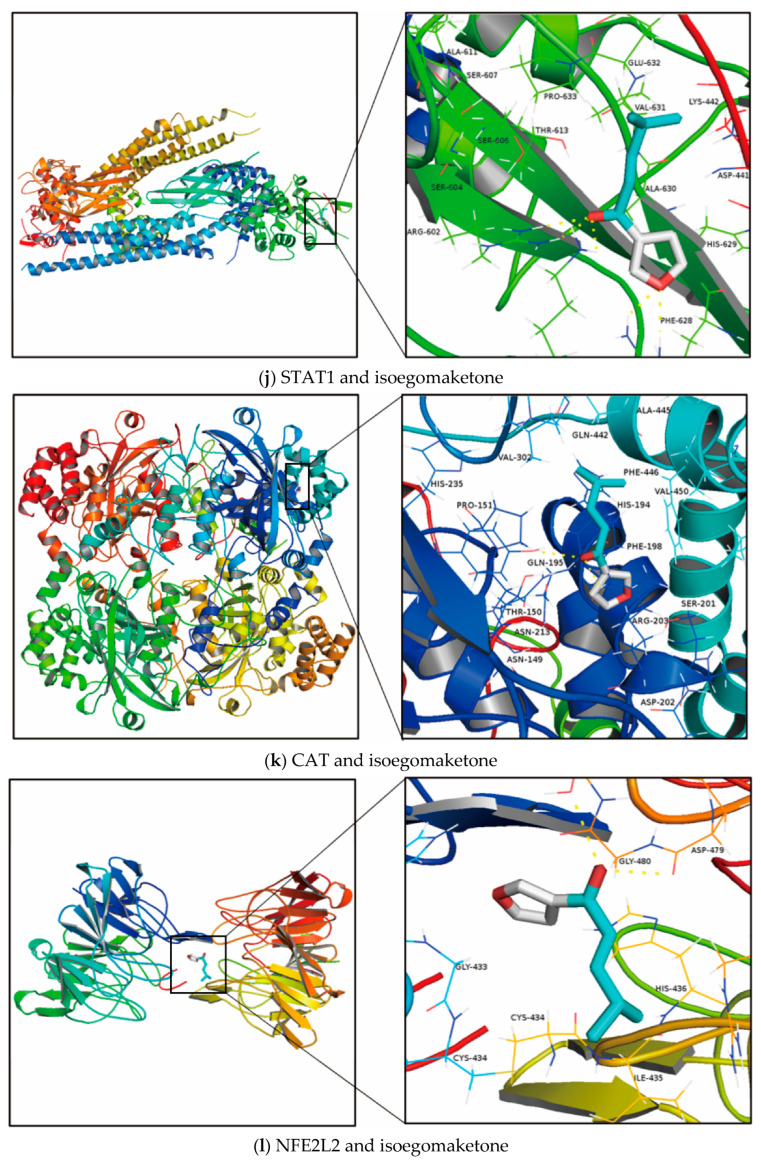
Images of the nine best docking results: (**a**) AKT1, (**b**) TP53, (**c**) JUN, (**d**) MAPK8, (**e**) CASP3, (**f**) IL6, (**g**) MTOR, (**h**) MAPK14, (**i**) CASP8, (**j**) STAT1, (**k**) CAT, (**l**) NFE2L2.

In general, the likelihood of interaction decreases with the energy of conformational stability of the ligand and receptor. There is yet no single standard for the target screening of active compounds, and, at present, binding energy ≤ −5.0 kcal/mol is typically used as the basis for screening. The findings of molecular docking revealed that 6 of the 12 chosen target proteins exhibited an affinity for isoegomaketone that was less than −5.0 kcal/mol. All docking results are shown in Table 4 and Figure 5.

## 4. Discussion

Currently, network pharmacology is being used with increasing frequency in the development and utilization of novel drugs. It converts the “single target, single drug” concept of drug research into a “network target, multicomponent treatment” model [35]. As a result, in this study, we built a multidimensional network through target prediction and protein interaction networks based on the network pharmacology and molecular docking research methodologies to clarify the probable targets of isoegomaketone.

Drug-likeness is recognized as a guideline for chemical library screening optimization in the area of drug development [36]. Lipinski’s rule of five, the Ghose filter, and Veber’s Rule are some of the most widely used rules of thumb. The main purpose of these tests is to determine whether a substance may be used as a drug or whether a substance, having pharmacological or biological activity, can be converted into an oral drug for human use. All of these formulas evaluate specific values of a given compound, including logP, TPSA, MW, MR, nHBA, nRotB, and nHBD. According to Table 1, isoegomaketone, with MW ≤ 500, logP < 5, nRotB ≤ 10, nHBD ≤ 5, nHBA ≤ 10, MR ≤ 140, and TPSA < 140 Å, perfectly passes the test of Lipinski’s rule of five, the Ghose filter, and Veber’s rule. Typically, a molecule that satisfies two of the three rules can be considered to fit the bioavailability of drugs. Thus, this study strongly indicates that isoegomaketone has excellent bioavailability in drugs, making it plausible that it is an active oral substance in humans. In addition, according to Table 1, isoegomaketone was predicted to have high gastrointestinal absorption, and it was also predicted to be able to permeate the blood–brain barrier. The LogS value for isoegomaketone is −2.39, which indicates its solubility in water. Moreover, isoegomaketone complies with the RO (3) rule and has good synthetic accessibility, which suggests its potential as a lead drug. The LD50 value of isoegomaketone was predicted to be 31.2 mg/kg, as shown in Table 2. According to DL-AOT, compounds are divided into four categories, namely “danger/poison”, “warning”, “caution”, and “none required”, and isoegomaketone is placed in the “caution” group, meaning that it is predicted to have no apparent toxicity to normal cells. All of these results indicate that isoegomaketone is likely to be a good candidate for drug development.

As indicated in Table 3, 48 possible isoegomaketone targets were found in the literature analysis. The STRING database was then used to examine the 48 possible targets, and there were 12 nodes whose degree value, betweenness centrality, and closeness centrality were all greater than the average, most of which (such as AKT1, TP53, JUN, MAPK8, CASP3, IL6, MTOR, MAPK14) are involved in the process of apoptosis and the stress response. These selected isoegomaketone targets suggest that isoegomaketone may have potential anti-inflammatory, anti-tumor, and cytotoxic activity. We also performed molecular docking to further investigate any potential interactions between isoegomaketone and these targets. With a decrease in the conformational energy of the ligand binding to the receptor, there is a higher chance of interaction. If −5.0 kcal/mol is utilized as the screening criterion, isoegomaketone may interact directly with these targets, since 6 out of the 12 targets had binding energies that were less than −5.0 kcal/mol [37,38,39,40].

Most of them are associated with anti-inflammatory, anti-tumor, and cytotoxic responses. They might, thus, be the main targets of isoegomaketone’s pharmacological activity.

To elucidate the potential biological activity of isoegomaketone based on gene functions and signaling pathways, we further conducted GO analysis and KEGG pathway analysis of these key targets with the clusterProfiler R package 3.8.1. According to the analysis of BP items, isoegomaketone is closely associated with the following: neuron death, regulation of neuron death, cellular response to chemical stress, aging, response to radiation, cellular response to abiotic stimulus, etc. The results of KEGG pathway enrichment analysis show that the lipid and atherosclerosis pathway is highly enriched. It should be mentioned that no previous studies have clarified the relationship between isoegomaketone and atherosclerosis. The result indicates that isoegomaketone may play a comprehensive role in preventing and treating atherosclerosis, as pathways such as the lipid and atherosclerosis pathway and fluid shear stress and atherosclerosis pathway are involved. In addition, the anti-inflammatory activity of isoegomaketone may also be involved. Further in vitro and in vivo experimental validation could be carried out. Moreover, we found that 9 of the top 20 enriched KEGG pathways were related to bacterial, viral, and parasite infections, such as hepatitis B, Kaposi sarcoma-associated herpesvirus, Epstein–Barr virus, human cytomegalovirus, human immunodeficiency virus 1, tuberculosis, Chagas disease (American trypanosomiasis), measles, and toxoplasmosis. According to these findings, isoegomaketone could possess antiviral, antibacterial, and parasitic action. Some pathways are related to inflammatory diseases—for example, the AGE-RAGE signaling pathway in non-alcoholic fatty liver disease and diabetic complications—which is in accordance with a previous in vivo study [9]. In addition, the KEGG enrichment results also include the TNF signaling pathway, Toll-like receptor signaling pathway, C-type lectin receptor signaling pathway, and other immuno-inflammatory-response-related pathways. As a result, our research revealed that isoegomaketone may be involved by preventing an overactive immune response, an influx of inflammatory substances, and the growth of tumor cells. These findings are similar to some of the in vitro studies of isoegomaketone that have reported its anti-inflammatory, anti-tumor, and cytotoxic activity [41]. Therefore, on one hand, it suggests the accuracy and rationality of our GO and KEGG research. On the other hand, the result further reveals the anti-inflammatory, anti-tumor, and cytotoxic activity target of isoegomaketone, suggesting the potential of isoegomaketone as an anti-inflammatory, anti-tumor, and cytotoxic drug. Taking these results together, we created a drug–target pathway network diagram that more obviously showed that isoegomaketone could exert a variety of pharmacological effects.

However, there are limitations to our research. The most significant is that, as mentioned above, our target screening was obtained through a large number of previously published studies. These provide high-quality, real-world evidence, based, in part, on in vivo and in vitro studies [5,6,16,17,18,19,20,21,22,23,24]. However, although our study provides some prospective evidence through the in silico method, some results have not yet been suitably empirically validated. Therefore, real-world validation using in vivo or in vitro methods based on the current prospective results is highly desirable.

## 5. Conclusions

Our research revealed that isoegomaketone has a wide range of pharmacological effects. At the same time, we investigated isoegomaketone’s potential targets, which may be used to further create secure and efficient anti-atherosclerosis, anti-inflammatory, anti-tumor, cytotoxic, antiviral, antibacterial, and anti-parasite medications. Our work offers a new perspective on the study, creation, and clinical use of isoegomaketone.

## Figures and Tables

**Table 1:** In silico drug properties of isoegomaketone.

MW (g/mol)	LogP	nRotB	nHBD	nHBA	MR	TPSA (Å^2^)	LogS	GI Absorption	BBB Permeance
164.20	2.40	3	0	2	47.7	30.21	−2.39	High	Yes
**Lipinski**	**Ghose**	**Veber**	**RO(3)**	**Synthetic accessibility**					
Yes	Yes	Yes	Yes	2.89					

MW = molecular weight, LogP = Log Po/w (iLOGP), nRotB = number of rotatable bonds, nHBD = number of H-bond donors, nHBA = number of H-bond acceptors, MR = molar refractivity, TPSA = topological polar surface area, LogS = Log S (ESOL), GI absorption = gastrointestinal absorption, BBB = blood–brain barrier.

**Table 2:** LD50 values of isoegomaketone.

Compound	LD50	Toxicity Evaluation
isoegomaketone	31.2	Caution

Toxicity evaluation classification: (1) danger/poison; (2) warning; (3) caution; (4) none required.

**Table 3:** Candidate target genes of isoegomaketone [5,6,16,17,18,19,20,21,22,23,24].

No.	Gene ID	Gene Name	Gene Description
1	ENSG00000275199	AKT3	AKT serine/threonine kinase 3
2	ENSG00000171791	BCL2	BCL2 apoptosis regulator
3	ENSG00000015475	BID	BH3 interacting domain death agonist
4	ENSG00000156709	AIFM1	apoptosis inducing factor mitochondria associated 1
5	ENSG00000121691	CAT	catalase
6	ENSG00000188130	MAPK12	mitogen-activated protein kinase 12
7	ENSG00000185386	MAPK11	mitogen-activated protein kinase 11
8	ENSG00000077150	NFKB2	nuclear factor kappa B subunit 2
9	ENSG00000198793	MTOR	mechanistic target of rapamycin kinase
10	ENSG00000125740	FOSB	FosB proto-oncogene, AP-1 transcription factor subunit
11	ENSG00000172115	CYCS	cytochrome c, somatic
12	ENSG00000007171	NOS2	nitric oxide synthase 2
13	ENSG00000104856	RELB	RELB proto-oncogene, NF-kB subunit
14	ENSG00000132906	CASP9	caspase 9
15	ENSG00000105221	AKT2	AKT serine/threonine kinase 2
16	ENSG00000243955	GSTA1	glutathione S-transferase alpha 1
17	ENSG00000181019	NQO1	NAD(P)H quinone dehydrogenase 1
18	ENSG00000123080	CDKN2C	cyclin dependent kinase inhibitor 2C
19	ENSG00000111537	IFNG	interferon gamma
20	ENSG00000105851	PIK3CG	phosphatidylinositol-4,5-bisphosphate 3-kinase catalytic subunit gamma
21	ENSG00000142208	AKT1	AKT serine/threonine kinase 1
22	ENSG00000112062	MAPK14	mitogen-activated protein kinase 14
23	ENSG00000156711	MAPK13	mitogen-activated protein kinase 13
24	ENSG00000213366	GSTM2	glutathione S-transferase mu 2
25	ENSG00000130522	JUND	JunD proto-oncogene, AP-1 transcription factor subunit
26	ENSG00000120889	TNFRSF10B	TNF receptor superfamily member 10b
27	ENSG00000164305	CASP3	caspase 3
28	ENSG00000051382	PIK3CB	phosphatidylinositol-4,5-bisphosphate 3-kinase catalytic subunit beta
29	ENSG00000162924	REL	REL proto-oncogene, NF-kB subunit
30	ENSG00000115415	STAT1	signal transducer and activator of transcription 1
31	ENSG00000109320	NFKB1	nuclear factor kappa B subunit 1
32	ENSG00000141510	TP53	tumor protein p53
33	ENSG00000107643	MAPK8	mitogen-activated protein kinase 8
34	ENSG00000197448	GSTK1	glutathione S-transferase kappa 1
35	ENSG00000116044	NFE2L2	nuclear factor, erythroid 2 like 2
36	ENSG00000124762	CDKN1A	cyclin dependent kinase inhibitor 1A
37	ENSG00000127666	TICAM1	Toll-like receptor adaptor molecule 1
38	ENSG00000087088	BAX	BCL2-associated X, apoptosis regulator
39	ENSG00000173039	RELA	RELA proto-oncogene, NF-kB subunit
40	ENSG00000175197	DDIT3	DNA damage inducible transcript 3
41	ENSG00000121879	PIK3CA	phosphatidylinositol-4,5-bisphosphate 3-kinase catalytic subunit alpha
42	ENSG00000177606	JUN	Jun proto-oncogene, AP-1 transcription factor subunit
43	ENSG00000136244	IL6	interleukin 6
44	ENSG00000143799	PARP1	poly(ADP-ribose) polymerase 1
45	ENSG00000115966	ATF2	activating transcription factor 2
46	ENSG00000108691	CCL2	C-C motif chemokine ligand 2
47	ENSG00000171608	PIK3CD	phosphatidylinositol-4,5-bisphosphate 3-kinase catalytic subunit delta
48	ENSG00000064012	CASP8	caspase 8

**Table 4:** Compound–target molecular docking binding energy.

No.	Target	PDB ID	Binding Energy (kcal/mol)
1	AKT1	2uzs	−3.8
2	TP53	6shz	−5.2
3	JUN	1jun	−3.7
4	MAPK8	1ukh	−5.2
5	CASP3	1cp3	−5.3
6	IL6	1alu	−4.7
7	MTOR	1aue	−4.6
8	MAPK14	1a9u	−5.5
9	CASP8	1qtn	−5.4
10	STAT1	1yvl	−4.3
11	CAT	1dgf	−6.0
12	NFE2L2	3zgc	−3.8

## Data Availability

The data used to support the findings of this study are included within the article.

## Supplementary Materials

The following supporting information can be downloaded at: https://www.mdpi.com/article/10.3390/life12122115/s1, Table S1. Topological parameters of key targets in protein interaction network.

## References

[1] Xu H., Zhang Y., Wang P., Zhang J., Chen H., Zhang L., Du X., Zhao C., Wu D., Liu F. (2021). A comprehensive review of integrative pharmacology-based investigation: A paradigm shift in traditional Chinese medicine. Acta Pharm. Sin. B.

[2] Xu X. (2006). New concepts and approaches for drug discovery based on traditional Chinese medicine. Drug Discov. Today Technol..

[3] Başer K., Demirci B., Dönmez A. (2003). Composition of the essential oil of *Perilla frutescens* (L.) Britton from Turkey. Flavour Fragr. J..

[4] Ha T.J., Lee M.-H., Lee J.H. (2015). Comparison of antioxidant activities and volatile components using GC/MS from leaves of Korean purple perilla (Perilla frutescens) grown in a greenhouse. Food Sci. Biotechnol..

[5] Lee J.-H., Cho H.-D., Jeong I.-Y., Lee M.-K., Seo K.-I. (2014). Sensitization of tumor necrosis factor-related apoptosis-inducing ligand (TRAIL)-resistant primary prostate cancer cells by isoegomaketone from Perilla frutescens. J. Nat. Prod..

[6] Park Y.D., Jin C.H., Choi D.S., Byun M.-W., Jeong I.Y. (2011). Biological evaluation of isoegomaketone isolated from Perilla frutescens and its synthetic derivatives as anti-inflammatory agents. Arch. Pharmacal Res..

[7] Kim Y.-R., Nam B., Han A.-R., Kim J.-B., Jin C.H. (2021). Isoegomaketone from *Perilla frutescens* (L.) Britt stimulates MAPK/ERK pathway in human keratinocyte to promote skin wound healing. Evid.-Based Complement. Altern. Med..

[8] Jin C.H., So Y., Nam B., Han S.N., Kim J.-B. (2017). Isoegomaketone alleviates the development of collagen antibody-induced arthritis in male Balb/c mice. Molecules.

[9] So Y., Jo Y.H., Nam B.M., Lee S.Y., Kim J.-B., Kang S.-Y., Jeong H.G., Jin C.H. (2015). Anti-obesity effect of isoegomaketone isolated from *Perilla frutescens* (L.) Britt. cv. Leaves. Korean J. Pharmacogn..

[10] Guan M., Guo L., Ma H., Wu H., Fan X. (2021). Network pharmacology and molecular docking suggest the mechanism for biological activity of rosmarinic acid. Evid.-Based Complement. Altern. Med..

[11] Li H., Hung A., Yang A.W.H. (2021). Herb-target virtual screening and network pharmacology for prediction of molecular mechanism of Danggui Beimu Kushen Wan for prostate cancer. Sci. Rep..

[12] Hopkins A.L. (2008). Network pharmacology: The next paradigm in drug discovery. Nat. Chem. Biol..

[13] Chen C.Y.-C. (2011). TCM Database@ Taiwan: The world’s largest traditional Chinese medicine database for drug screening in silico. PLoS ONE.

[14] Daina A., Michielin O., Zoete V. (2017). SwissADME: A free web tool to evaluate pharmacokinetics, drug-likeness and medicinal chemistry friendliness of small molecules. Sci. Rep..

[15] Xu Y., Pei J., Lai L. (2017). Deep learning based regression and multiclass models for acute oral toxicity prediction with automatic chemical feature extraction. J. Chem. Inf. Model..

[16] Wang Y. (2013). Exploration of the Effect and Mechanism of Radiosensitization of Isoegomaketone on Hepatocellular Carcinoma Cells. Ph.D. Thesis.

[17] Wu G., Huan X., Wu X., Tian J., Liu J., Liu F., Yao X. (2020). The preliminary study for radiotherapy sensitization effect of isoegomaketone on human colorectal cancer xenograft tumor in nude mice. J. Multidiscip. Cancer Manag. (Electron. Version).

[18] Yang F.C., Wang Y.J. (2016). The effect of radiosensitization of isoegomaketone on lung cancer cells and the involvement of endoplasmic reticulum stress. J. Clin. Exp. Med..

[19] Cho B.O., Jin C.H., Park Y.D., Ryu H.W., Byun M.W., Seo K.I., Jeong I.Y. (2011). Isoegomaketone induces apoptosis through caspase-dependent and caspase-independent pathways in human DLD1 cells. Biosci. Biotechnol. Biochem..

[20] Kwon S.-J., Lee J.-H., Moon K.-D., Jeong I.-Y., Ahn D.-U., Lee M.-K., Seo K.-I. (2014). Induction of apoptosis by isoegomaketone from Perilla frutescens L. in B16 melanoma cells is mediated through ROS generation and mitochondrial-dependent,-independent pathway. Food Chem. Toxicol..

[21] Kwon S.-J., Lee J.-H., Moon K.-D., Jeong I.-Y., Yee S.-T., Lee M.-K., Seo K.-I. (2014). Isoegomaketone induces apoptosis in SK-MEL-2 human melanoma cells through mitochondrial apoptotic pathway via activating the PI3K/Akt pathway. Int. J. Oncol..

[22] Wang Y., Huang X., Han J., Zheng W., Ma W. (2013). Extract of Perilla frutescens inhibits tumor proliferation of HCC via PI3K/AKT signal pathway. Afr. J. Tradit. Complement. Altern. Med..

[23] Jin C.H., Lee H.J., Park Y.D., Choi D.S., Kim D.S., Kang S.Y., Seo K.I., Jeong I.Y. (2010). Isoegomaketone inhibits lipopolysaccharide-induced nitric oxide production in RAW 264.7 macrophages through the heme oxygenase-1 induction and inhibition of the interferon-beta-STAT-1 pathway. J. Agric. Food Chem..

[24] Jin C.H., So Y.K., Han S.N., Kim J.B. (2016). Isoegomaketone Upregulates Heme Oxygenase-1 in RAW264.7 Cells via ROS/p38 MAPK/Nrf2 Pathway. Biomol. Ther..

[25] Szklarczyk D., Gable A.L., Lyon D., Junge A., Wyder S., Huerta-Cepas J., Simonovic M., Doncheva N.T., Morris J.H., Bork P. (2018). STRING v11: Protein–protein association networks with increased coverage, supporting functional discovery in genome-wide experimental datasets. Nucleic Acids Res..

[26] Li X., Wei S., Niu S., Ma X., Li H., Jing M., Zhao Y. (2022). Network pharmacology prediction and molecular docking-based strategy to explore the potential mechanism of Huanglian Jiedu Decoction against sepsis. Comput. Biol. Med..

[27] Chin C.H., Chen S.H., Wu H.H., Ho C.W., Ko M.T., Lin C.Y. (2014). cytoHubba: Identifying hub objects and sub-networks from complex interactome. BMC Syst. Biol..

[28] The Gene Ontology Consortium (2017). Expansion of the Gene Ontology knowledgebase and resources. Nucleic Acids Res..

[29] Kanehisa M., Furumichi M., Tanabe M., Sato Y., Morishima K. (2017). KEGG: New perspectives on genomes, pathways, diseases and drugs. Nucleic Acids Res..

[30] Lipinski C.A., Lombardo F., Dominy B.W., Feeney P.J. (2001). Experimental and computational approaches to estimate solubility and permeability in drug discovery and development settings. Adv. Drug Deliv. Rev..

[31] Ghose A.K., Viswanadhan V.N., Wendoloski J.J. (1999). A knowledge-based approach in designing combinatorial or medicinal chemistry libraries for drug discovery. 1. A qualitative and quantitative characterization of known drug databases. J. Comb. Chem..

[32] Veber D.F., Johnson S.R., Cheng H.Y., Smith B.R., Ward K.W., Kopple K.D. (2002). Molecular properties that influence the oral bioavailability of drug candidates. J. Med. Chem..

[33] Congreve M., Carr R., Murray C., Jhoti H. (2003). A ‘rule of three’ for fragment-based lead discovery?. Drug Discov. Today.

[34] Fukunishi Y., Kurosawa T., Mikami Y., Nakamura H. (2014). Prediction of synthetic accessibility based on commercially available compound databases. J. Chem. Inf. Model..

[35] Zhang R., Zhu X., Bai H., Ning K. (2019). Network Pharmacology Databases for Traditional Chinese Medicine: Review and Assessment. Front. Pharmacol..

[36] Di L., Kerns E.H., Carter G.T. (2009). Drug-like property concepts in pharmaceutical design. Curr. Pharm. Des..

[37] Zhuang Y., Zhang X., Luo S., Wei F., Song Y., Lin G., Yao M., Gong A. (2022). Exploring the Molecular Mechanism of Zhi Bai Di Huang Wan in the Treatment of Systemic Lupus Erythematosus Based on Network Pharmacology and Molecular Docking Techniques. Processes.

[38] Huang X., Rehman H.M., Szöllősi A.G., Zhou S. (2022). Network Pharmacology-Based Approach Combined with Bioinformatic Analytics to Elucidate the Potential of Curcumol against Hepatocellular Carcinoma. Genes.

[39] Dai L., Zhang G., Wan X. (2022). Network Pharmacology and Molecular Docking Analysis Reveal Insights into the Molecular Mechanism of Shengma-Gegen Decoction on Monkeypox. Pathogens.

[40] Zhou W., Wang J., Wu Z., Huang C., Lu A., Wang Y. (2016). Systems pharmacology exploration of botanic drug pairs reveals the mechanism for treating different diseases. Sci. Rep..

[41] Wang R., Zhang Q., Feng C., Zhang J., Qin Y., Meng L. (2022). Advances in the Pharmacological Activities and Effects of Perilla Ketone and Isoegomaketone. Evid.-Based Complement. Alternat. Med..

