# Supplementary material for: Defining the Potential Targets for Biological Activity of Isoegomaketone Based on Network Pharmacology and Molecular Docking Methods
Source: Life (Basel). 2022 Dec 15;12(12):2115. doi: 10.3390/life12122115 (PMC9788221; doi:10.3390/life12122115)
Supplement: Supplementary file 1 [file life-12-02115-s001.zip › life-2051622-supplementary.docx]

**Supplementary Materials**

Defining the potential targets for biological activity of isoegomaketone based on network pharmacology and molecular docking methods

**Juzhao Zhang^1^, Ruo Wang^1,*^, Yuxuan Qin and Chengling Feng**

Shanghai Jiao Tong University School of Medicine, Shanghai 200025, China

1 These authors contributed equally to this work.

*Correspondence: author: wangruo@sjtu.edu.cn

**Table S1.** Topological parameters of key targets in protein interaction network.

| **Name** | **Degree** | **Betweenness Centrality** | **Closeness Centrality** |
| --- | --- | --- | --- |
| AKT1 | 41 | 0.08105111 | 0.88679245 |
| TP53 | 41 | 0.0741373 | 0.88679245 |
| JUN | 38 | 0.07318986 | 0.83928571 |
| MAPK8 | 35 | 0.04229935 | 0.79661017 |
| CASP3 | 34 | 0.02992914 | 0.78333333 |
| IL6 | 32 | 0.03697473 | 0.75806452 |
| MTOR | 30 | 0.02808919 | 0.734375 |
| MAPK14 | 30 | 0.02083125 | 0.734375 |
| CASP8 | 29 | 0.02981882 | 0.72307692 |
| STAT1 | 25 | 0.01965111 | 0.68115942 |
| CAT | 25 | 0.04435014 | 0.68115942 |
| NFE2L2 | 22 | 0.02159998 | 0.65277778 |
